# Integrated Analysis of DNA Methylation and mRNA Expression Profiles Data to Identify Key Genes in Lung Adenocarcinoma

**DOI:** 10.1155/2016/4369431

**Published:** 2016-08-17

**Authors:** Xiang Jin, Xingang Liu, Xiaodan Li, Yinghui Guan

**Affiliations:** ^1^Department of Respiration, The First Hospital of Jilin University, Changchun 130021, China; ^2^ICU Department, The First Hospital of Jilin University, Changchun 130021, China

## Abstract

*Introduction*. Lung adenocarcinoma (LAC) is the most frequent type of lung cancer and has a high metastatic rate at an early stage. This study is aimed at identifying LAC-associated genes.* Materials and Methods*. GSE62950 downloaded from Gene Expression Omnibus included a DNA methylation dataset and an mRNA expression profiles dataset, both of which included 28 LAC tissue samples and 28 adjacent normal tissue samples. The differentially expressed genes (DEGs) were screened by Limma package in R, and their functions were predicted by enrichment analysis using TargetMine online tool. Then, protein-protein interaction (PPI) network was constructed using STRING and Cytoscape. Finally, LAC-associated methylation sites were identified by CpGassoc package in R and mapped to the DEGs to obtain LAC-associated DEGs.* Results*. Total 913 DEGs were identified in LAC tissues. In the PPI networks,* MAD2L1*,* AURKB*,* CCNB2*,* CDC20,* and* WNT3A* had higher degrees, and the first four genes might be involved in LAC through interaction. Total 8856 LAC-associated methylation sites were identified and mapped to the DEGs. And there were 29 LAC-associated methylation sites located in 27 DEGs (e.g.,* SH3GL2*,* BAI3*,* CDH13*,* JAM2*,* MT1A*,* LHX6,* and* IGFBP3*).* Conclusions*. These key genes might play a role in pathogenesis of LAC.

## 1. Introduction

As the most common type of lung cancer and a non-small cell lung carcinoma (NSCLC) [1], lung adenocarcinoma (LAC) is characterized by gland or duct formation and massive mucus production [2]. LAC generally originates in peripheral lung tissue, and this is in contrast with squamous cell lung cancer and small cell lung cancer (SCLC), which are both apt to be located more centrally in lungs [3, 4]. In US, LAC accounts for approximately 40% of lung cancers [5]. Smoking is the main cause of LAC, and the disease has a high metastasis rate even at an early stage [4]. Therefore, it is necessary to identify key genes in LAC and develop effective therapeutic schedule.

The WNT/TCF signaling can promote osseous and brain metastasis of LAC cells through targeting* HOXB9* and* LEF1 *which mediates chemotactic invasion and colony outgrowth [6]. Coexpression of* Oct4* and* Nanog*, which are homeobox transcription factors important for self-renewal of stem cells, commands epithelial-mesenchymal transdifferentiation, mediates tumor-initiating ability, and contributes to metastasis of LAC [7]. As a noncoding RNA,* MALAT-1* enhances motility of LAC cells via regulating motility-associated gene expression in transcriptional and posttranscriptional levels [8, 9].* BRAF* mutation is frequently detected in human LAC, indicating that* BRAF* may serve as a therapeutic target for a subset of patients with the disease [10, 11]. Overexpression of* caveolin-1* is essential for mediating filopodia formation, which may promote invasion of LAC cells [12]. In spite of the above researches, the mechanisms of LAC still remain unclear.

Recently, along with the development of chip technology, microarray data have been obtained and uploaded to Gene Expression Omnibus (GEO) for us to study [13]. Using microarray data GSE62950, we screened the differentially expressed genes (DEGs) between LAC tissue samples and adjacent normal tissue samples. And their potential functions were predicted by Gene Ontology (GO), Kyoto Encyclopedia of Genes and Genomes (KEGG), and Disease Ontology (DO) enrichment analysis. Then, the interrelationships between these DEGs were analyzed by protein-protein interaction (PPI) network and module analysis. At last, LAC-associated methylation sites were identified and mapped to the DEGs to obtain LAC-associated DEGs.

## 2. Materials and Methods

### 2.1. Microarray Data

Microarray data GSE62950 was downloaded from the database of GEO (http://www.ncbi.nlm.nih.gov/geo/), which included a DNA methylation dataset and an mRNA expression profiles dataset. The DNA methylation dataset and the mRNA expression profiles dataset separately were based on the platform of GPL8432 Illumina HumanRef-8 WG-DASL v3.0 and GPL8490 Illumina HumanMethylation27 BeadChip (HumanMethylation27_270596_v.1.2), and both of them included 28 LAC tissue samples and 28 adjacent normal tissue samples.

### 2.2. Data Preprocessing

Normalized series matrix file of mRNA expression profiles was downloaded directly. After beta matrix of DNA methylation data was downloaded, primary methylation signals were preprocessed by Methylation Module V1.9 [14] in BeadStudio V3.1.0.0 to obtain normalized beta matrix. Those methylation sites which had no signal values in one or more samples were removed.

### 2.3. DEGs Screening

Using Limma package [15] in R, the DEGs between LAC tissue samples and adjacent normal tissue samples were identified. The *p* values of DEGs were adjusted by Benjamini-Hochberg (BH) method [16]. The adjusted *p* value < 0.05 and |log_2_⁡fold-change(FC) | ≥1 were taken as the thresholds.

### 2.4. GO, KEGG, and DO Enrichment Analysis

GO is utilized for predicting potential functions of gene products in three categories (biological process, BP; cellular component, CC; and molecular function, MF) [17]. The KEGG database can be used for systematic analysis of gene functions, which connects genomic information with corresponding functional information [18]. The DO database contains a comprehensive knowledge of human diseases and is applied to annotate diseases [19]. Using TargetMine online tool [20], GO, KEGG, and DO enrichment analyses were performed for the DEGs. The *p* values of enriched terms were corrected by Holm-Bonferroni [21]. The adjusted *p* value < 0.05 was used as the cut-off criterion.

### 2.5. PPI Network and Module Construction

The STRING [22] database was utilized to search PPI pairs among the DEGs. And combined score > 0.4 was used as the cut-off criterion. Then, the PPI network of the DEGs was visualized by Cytoscape (http://www.cytoscape.org/) [23]. Subsequently, igraph package [24] in R was used to calculate connectivity degrees of nodes (proteins) in the PPI network, and nodes with higher degrees were taken as hub nodes.

Furthermore, MCODE plugin [25] in Cytoscape was used to screen modules from the PPI network. Using BinGO plugin [26], GO functional enrichment analysis was conducted for the genes in each module.

### 2.6. Screening of LAC-Associated Methylation Sites

Using genefilter package [27] in R, the methylation sites which had higher beta value variations within groups compared with variations among groups were deleted. And the *p* value < 0.05 was used as the cut-off criterion. Then, the methylation sites located in sex chromosomes were removed. Finally, the associations between the screened methylation sites and LAC were analyzed by CpGassoc package [28] in R. The *p* value < 0.05 was taken as the threshold.

### 2.7. Correlation Analysis of Methylation Sites and DEGs

According to the annotation information of the DNA methylation profiles, the nearest genes to the LAC-associated methylation sites were obtained and then mapped to the DEGs. At last, LAC-associated methylation sites of the DEGs were gained.

### 2.8. Validation of Methylation Sites and DEGs in LAC Tissue Samples

The RNASeqV2 data of LAC were downloaded from The Cancer Genome Atlas (TCGA, http://cancergenome.nih.gov/) database, which included 515 LAC tissue samples and 59 adjacent normal tissue samples. Using Limma package [15] in R, the DEGs with the adjusted *p* value < 0.05 and |log_2_FC| ≥ 1 were also screened. Meanwhile, the methylation data of LAC were also downloaded from TCGA database, which included 459 LAC tissue samples and 32 adjacent normal tissue samples. Moreover, LAC-associated methylation sites were also identified and mapped to the DEGs using the same methods as the above.

## 3. Results

### 3.1. Data Preprocessing and DEGs Screening

**Figure 1:**
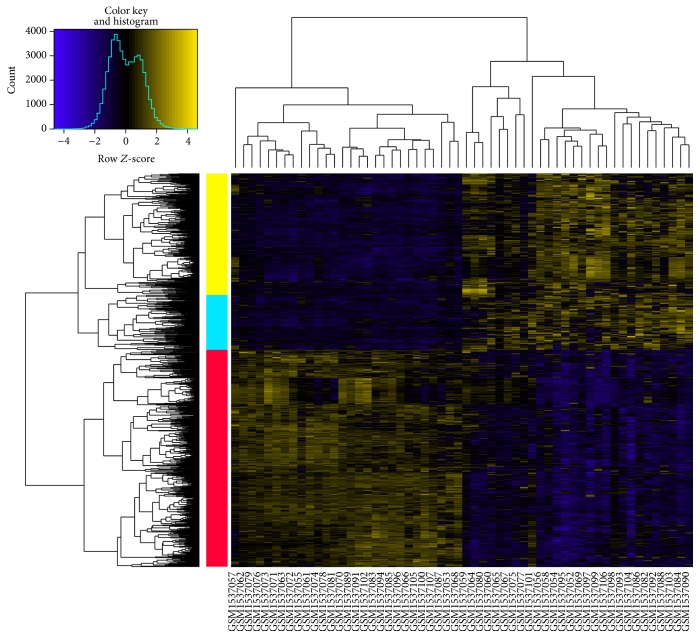
The heat map of the DEGs. Yellow and blue bars represent upregulated and downregulated genes, respectively. Two-way clustering results in the left side indicate that DEGs were clustered into three categories. Yellow and blue columns represent upregulated genes in lung adenocarcinoma tissues, while red column stands for downregulated genes.

After preprocessing, total 18626 beta values of DNA methylation data and 18626 gene expression values of mRNA expression profiles separately were obtained. Compared with adjacent normal tissue samples, there were a total of 913 DEGs (including 409 upregulated and 504 downregulated genes) in LAC tissue samples. In the heat map of the DEGs, LAC tissues could be definitely separated from adjacent normal tissues by the DEGs (Figure 1).

### 3.2. GO, KEGG, and DO Enrichment Analysis

Using TargetMine online tool, functions of the DEGs were predicted by GO, KEGG, and DO enrichment analyses. For the upregulated genes, the enriched GO functions included multicellular organismal catabolic process (*p* value = 8.59*E* − 07) in BP category, as well as extracellular region (*p* value = 2.67*E* − 04) and extracellular space (*p* value = 0.002024) in CC category (Table 1(a)). The enriched KEGG pathways for upregulated genes included protein digestion and absorption (*p* value = 0.001244) and cell cycle (*p* value = 0.026338, which involved cell division cycle 20,* CDC20*; cyclin B2,* CCNB2*; and mitotic arrest deficient 2-like 1,* MAD2L1*) (Table 1(b)). The enriched DO terms for upregulated genes included cell type cancer (adjust. *p* value = 6.12*E* − 05), germ cell cancer (adjust. *p* value = 0.001326), embryoma (adjust. *p* value = 0.008727), and embryonal cancer (adjust. *p* value = 0.009481) (Table 1(c)). And all of the DO terms involved* MAD2L1* and aurora B kinase (*AURKB*). The enriched GO functions for downregulated genes were listed in Table 1(d), including single-organism process (*p* value = 5.73*E* − 05) and single-multicellular organism process (*p* value = 1.93*E* − 04, which involved wingless-related MMTV integration site 3A,* WNT3A*) in BP category, as well as cell periphery in CC category (*p* value = 1.03*E* − 05, which involved* WNT3A*). In addition, there were nonsignificant KEGG pathways and DO terms enriched for the downregulated genes.

### 3.3. PPI Network and Module Analysis

**Figure 2:**
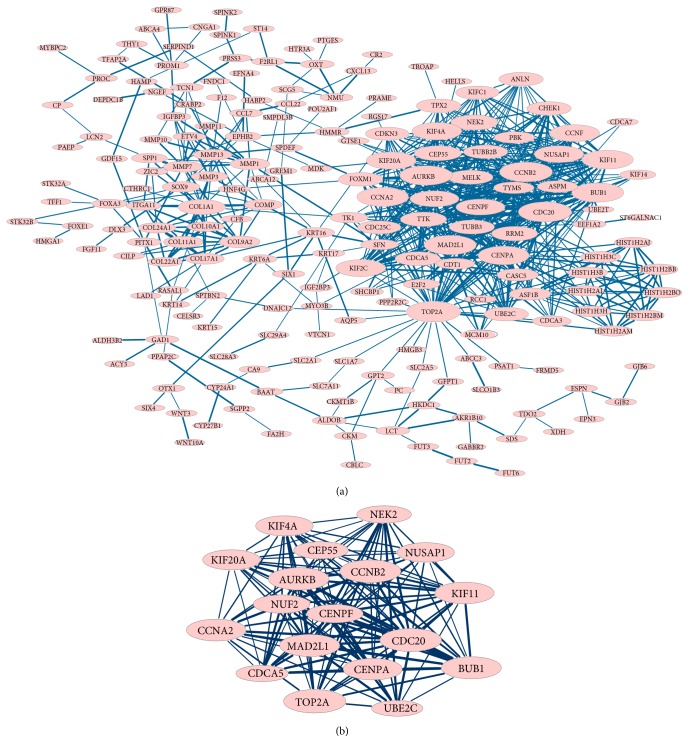
The PPI network and module for upregulated genes. (a) The PPI network for upregulated genes. (b) Module 1 of the PPI network for upregulated genes. Red nodes represent upregulated genes. Thickness of edges is in direct proportion to combined scores. Node sizes are positively correlated with connectivity degrees of nodes.

PPI networks for upregulated and downregulated genes were constructed, respectively. The PPI network for upregulated genes had 229 nodes and 725 interactions (Figure 2(a)). Particularly,* MAD2L1* (degree = 36),* AURKB* (degree = 38),* CCNB2* (degree = 40), and* CDC20* (degree = 42) had higher degrees, and they can interact with each other in the PPI network. Several modules were screened from the PPI network for upregulated genes, and the largest module (module 1) is also showed in Figure 2(b). GO functional enrichment analysis showed that the genes in module 1 were involved in mitosis-associated terms.

**Figure 3:**
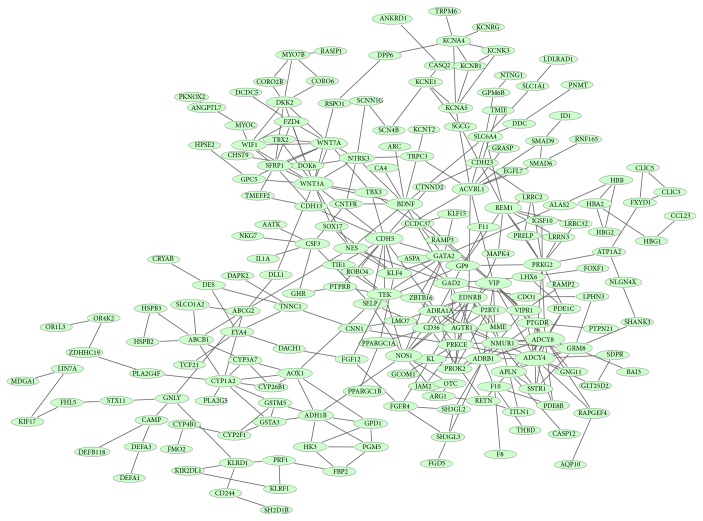
The PPI network for downregulated genes. Green nodes represent downregulated genes. Thickness of edges is in direct proportion to combined scores. Node sizes are positively correlated with connectivity degrees of nodes.

The PPI network for downregulated genes had 233 nodes and 368 interactions (Figure 3). Connectivity degrees of the nodes in the PPI network were calculated, and cGMP-dependent protein kinase II (*PRKG2*, degree = 11), VE-cadherin (*CDH5*, degree = 12),* WNT3A* (degree = 15), adenylyl cyclase 8 (*ADCY8*, degree = 16), and adenylyl cyclase 4 (*ADCY4*, degree = 17) were the top 5 nodes which had higher degrees. What is more, nonsignificant modules were screened from the PPI network for downregulated genes.

### 3.4. Screening of LAC-Associated Methylation Sites

After screening, total 8856 methylation sites were obtained. Then, the associations between the screened methylation sites and LAC were analyzed under the threshold of *p* value < 0.05. As a result, 230 LAC-associated methylation sites were identified.

### 3.5. Correlation Analysis of Methylation Sites and DEGs

There were 29 LAC-associated methylation sites located in 27 DEGs (e.g., Src homology 3 domain GRB2-like 2,* SH3GL2*; brain-specific angiogenesis inhibitor 3,* BAI3*; H-cadherin,* CDH13*; junctional adhesion molecule 2,* JAM2*; metallothionein 1A,* MT1A*; LIM-homeobox containing 6,* LHX6*; and insulin-like growth factor binding protein-3,* IGFBP3*) (Table 2). And all of the methylation sites were within 2 kb from transcriptional start sites (TSSs) of the DEGs. For the 29 methylation sites, their methylation indexes in LAC tissue samples were compared with that in adjacent normal tissue samples. The methylation indexes of 19 methylation sites were significantly increased, and the downstream genes mediated by those 19 methylation sites were downregulated. Nevertheless, 1 methylation site had significantly decreased methylation index, and its downstream genes were upregulated.

### 3.6. Validation of Methylation Sites and DEGs in LAC Tissue Samples

Total 4893 DEGs (including 2191 upregulated genes and 2702 downregulated genes) were identified in the LAC sample data downloaded from TCGA database. There were 691 common DEGs (including 310 upregulated genes and 381 downregulated genes) between the 4893 DEGs identified in the LAC sample data downloaded from TCGA database and the 913 DEGs identified in the microarray data of GSE62950. The common DEGs included genes such as* WNT3A*,* MAD2L1*,* AURKB*,* CCNB2*,* CDC20*,* SH3GL2*,* BAI3*,* CDH13*,* JAM2*,* MT1A,* and* IGFBP3*. In addition, the 29 LAC-associated methylation sites identified in the microarray data of GSE62950 were also detected in the methylation data downloaded from TCGA database.

## 4. Discussion

In this study, 913 DEGs including 409 upregulated and 504 downregulated genes were identified in LAC tissue samples compared with adjacent normal tissue samples. Total 230 LAC-associated methylation sites were identified, among which 29 LAC-associated methylation sites were located in 27 DEGs. Afterwards, the RNASeqV2 data and methylation data of LAC were downloaded from TCGA database to validate the obtained methylation sites and DEGs. There were 691 common DEGs (such as* WNT3A*,* MAD2L1*,* AURKB*,* CCNB2*,* CDC20*,* SH3GL2*,* BAI3*,* CDH13*,* JAM2*,* MT1A,* and* IGFBP3*) between the 913 DEGs identified in the microarray data of GSE62950 and the 4893 DEGs identified in the LAC sample data downloaded from TCGA database. In addition, the 29 LAC-associated methylation sites identified in the microarray data of GSE62950 were also detected in the methylation data downloaded from TCGA database. Functional enrichment indicated that* WNT3A* was involved in single-multicellular organism process and cell periphery. Overexpression of* Wnt5a*, which belongs to* Wnt* family that encodes signaling glycoproteins, promotes invasion of NSCLC during tumor progression [29, 30]. Via activating JNK pathway,* Wnt-7a* and* Fzd-9 *signaling plays role in inducing the receptor tyrosine kinase inhibitor Sprouty-4 and cadherin proteins and is essential for maintaining epithelial differentiation and inhibiting transformed cell growth in some NSCLC patients [31]. In the PPI network for downregulated genes, WNT3A had higher degrees. These suggested that WNT3A might play a role in LAC.

Besides,* CDC20*,* CCNB2,* and* MAD2L1* were enriched in the pathway of cell cycle. Meanwhile,* MAD2L1* and* AURKB* were involved in DO terms of type cancer, germ cell cancer, embryoma, and embryonal cancer. Results of immunohistochemistry suggest that* CDC20* can be a negative marker in prognosis of patients with resected NSCLC, especially adenocarcinoma [32]. Overexpressed* CDK5RAP3* and* CCNB2*, as well as suppressed RAGE, may be promising biomarkers in lung adenocarcinoma [33]. The 5-year overall survival rates of LAC patients with low* CCNB2* mRNA levels were significantly higher than that with high levels, and overexpressed* CCNB2* mRNA can independently predict a poor prognosis in patients with LAC [34, 35]. Immunohistochemical analysis indicates* AURKB*, which mediates chromosome segregation during mitosis, is frequently overexpressed in primary lung carcinomas [36, 37]. Immunohistochemistry shows that overexpression of cell division cycle associated 8 (*CDCA8*) and* AURKB* can result in bad outcome of lung cancer patients; thus, suppression of the CDCA8-AURKB pathway is a potential therapeutic strategy for lung cancer [38]. Semiquantitative RT-PCR shows that mitotic arrest defective protein 2 (*MAD2*) is overexpressed in a high percentage of lung cancers, and multivariate analysis suggests that high-level* MAD2* can be a prognostic marker independently [39]. In the PPI network for upregulated genes,* MAD2L1*,* AURKB*,* CCNB2,* and* CDC20* had higher degrees, and they can interact with each other. Therefore,* MAD2L1*,* AURKB*,* CCNB2,* and* CDC20* might be implicated in LAC by interacting with each other.

Additionally, LAC-associated methylation sites were identified and mapped to the DEGs. And there were 29 LAC-associated methylation sites located in 27 DEGs (e.g.,* SH3GL2*,* BAI3*,* CDH13*,* JAM2*,* MT1A*,* LHX6,* and* IGFBP3*). Loss of* SH3GL2* is frequently detected in NSCLC and* SH3GL2* can mediate cellular growth and invasion through interacting with* EGFR* [40].* CDX2*,* VIL1,* and* BAI3 *levels have significant differences in SCLC and large-cell neuroendocrine lung carcinoma (LCNEC); therefore, they can be diagnostic markers of these tumor types [41]. Tumor suppressor gene* CDH13*, located on chromosome 16q24.2–3, is downregulated in lung cancer and its aberrant methylation may be a potential marker for cancer detection [42–44]. Via mediating *β*1 integrin subunit and ERK activation in human dermal lymphatic endothelial cells (HDLEC), junctional adhesion molecule-C (*JAM-C*) contributes to lymphangiogenesis and nodal metastasis, suggesting that* JAM-C* may be a target for treating lymphatic metastases in NSCLC [45]. Overexpression of metallothionein (*MT*) can be used as an independent predictor of short-term survival in SCLC patients enduring chemotherapy [46, 47]. Previous study indicates that* LHX6* is a candidate tumor suppressor gene that has epigenetic silencing in patients with lung cancer [48]. In NSCLC, methylation status of* IGFBP-3* before cisplatin therapy seems to be a biomarker of prognosis, helping to select appropriate therapeutic method for patients [49, 50]. These declared that* SH3GL2*,* BAI3*,* CDH13*,* JAM2*,* MT1A*,* LHX6,* and* IGFBP3* might relate to LAC.

In conclusion, we carried out a comprehensive bioinformatics analysis to screen LAC-associated genes. We identified 913 DEGs and 8856 methylation sites in LAC tissue samples. Besides, LAC might correlate with several key genes (e.g.,* WNT3A*,* MAD2L1*,* AURKB*,* CCNB2*,* CDC20*,* SH3GL2*,* BAI3*,* CDH13*,* JAM2*,* MT1A*,* LHX6,* and* IGFBP3*). However, these bioinformatic findings need to be validated by further researches.

## Figures and Tables

**(a):** 

Category	ID	Description	Count	Gene symbol	*p* value
BP	GO:0044243	Multicellular organismal catabolic process	15	*ACE2*, *COL10A1*, *COL11A1*,…	8.59*E* − 07
BP	GO:0030574	Collagen catabolic process	14	*FAP*, *MMP1*, *MMP10*,…	4.49*E* − 06
BP	GO:1903047	Mitotic cell cycle process	41	*ANLN*, *AURKB*, *BUB1*,…	1.70*E* − 05
BP	GO:0044259	Multicellular organismal macromolecule metabolic process	15	*ACE2*, *COL10A1*, *COL11A1*,…	4.16*E* − 05
BP	GO:0007067	Mitotic nuclear division	22	*ANLN*, *AURKB*, *CDC20*,…	4.57*E* − 05
CC	GO:0005576	Extracellular region	122	*ACE2*, *ACOT11*, *ACY3*,…	2.67*E* − 04
CC	GO:0005615	Extracellular space	46	*ACE2*, *AGR2*, *BPIFA1*,…	0.002024

**(b):** 

ID	Description	Count	Gene symbol	*p* value
hsa04974	Protein digestion and absorption	11	*ACE2*, *COL10A1*, *COL11A1*,…	0.001244
hsa04110	Cell cycle	11	*CDC20*, *MAD2L1*, *CCNB2*,…	0.026338

**(c):** 

DOID	Description	Count	Gene symbol	Adjust. *p* value
DOID:0050687	Cell type cancer	64	*AURKB*, *MAD2L1*, *ASPM*,…	6.12*E* − 05
DOID:2994	Germ cell cancer	41	*AURKB*,* MAD2L1*, *CA9*,…	0.001326
DOID:4766	Embryoma	38	*AURKB*, *BUB1*, *MAD2L1*,…	0.008727
DOID:688	Embryonal cancer	38	*AURKB*,* MAD2L1*, *CA9*,…	0.009481

**(d):** 

Category	ID	Description	Count	Gene symbol	*p* value
BP	GO:0044699	Single-organism process	296	*AADAC*, *ABCB1*, *ABCG2*,…	5.73*E* − 05
BP	GO:0044707	Single-multicellular organism process	150	*WNT3A*, *ACVRL1*, *ADCY4*,…	1.93*E* − 04
BP	GO:0003013	Circulatory system process	24	*ACVRL1*, *ADRA1A*, *ADRB1*,…	4.58*E* − 04
BP	GO:0032501	Multicellular organismal process	152	*WNT3A*, *ACVRL1*, *ADCY4*,…	0.001393
BP	GO:0008015	Blood circulation	23	*ACVRL1*, *ADRA1A*, *ADRB1*,…	0.001693
CC	GO:0031226	Intrinsic component of plasma membrane	72	*ACVRL1*, *ADRA1A*, *ADRB1*,…	2.38*E* − 06
CC	GO:0005886	Plasma membrane	139	*WNT3A*, *ADCY8*, *ADGRB3*,…	8.04*E* − 06
CC	GO:0071944	Cell periphery	140	*WNT3A*, *ANXA8*, *AQP10*,…	1.03*E* − 05
CC	GO:0044459	Plasma membrane part	86	*AGER*, *AGTR1*, *AQP10*,…	1.44*E* − 05
CC	GO:0005887	Integral component of plasma membrane	68	*FZD4*, *GHR*, *GP9*,…	1.75*E* − 05

**Table 2:** The 29 lung adenocarcinoma-associated methylation sites located in 27 DEGs.

IllumID	Chromosome	Meth.pos (Genome Build 36)	DEG	Distance_to_TSS	log_2_⁡FC⁡(DEG)	log_2_⁡FC⁡(β)
cg17398595	chr9	17568725	*SH3GL2*	469	−2.461984643	0.419097018
cg01817029	chr12	70951777	*TRHDE*	953	−2.080720357	0.379725667
cg18182399	chr2	219991419	*DES*	76	−1.912270714	0.648411911
cg01532771	chr19	7733560	*CLEC4M*	521	−1.867543929	0.109407145
cg17407908	chr6	32261093	*AGER*	1092	−1.805516071	−0.076347441
cg21057494	chr3	45041975	*CLEC3B*	799	−1.784051786	0.176075101
cg08555612	chr3	71917330	*PROK2*	428	−1.734814286	0.599490205
cg04884908	chr2	72228348	*CYP26B1*	123	−1.568955357	0.541885215
cg06615154	chr1	151789177	*S100A3*	819	−1.430114286	0.141081243
cg10244047	chr6	69402835	*BAI3*	1323	−1.417121429	0.364653419
cg18343862	chr11	10546579	*XLKD1*	276	−1.346532857	0.053465056
cg01880569	chr16	81217829	*CDH13*	250	−1.329384643	0.115405772
cg08977371	chr16	81217991	*CDH13*	88	−1.329384643	0.396503001
cg24829483	chr4	14950739	*C1QTNF7*	29	−1.192036429	0.119720772
cg08448751	chr3	52454641	*SEMA3G*	558	−1.143811071	0.044592487
cg03382304	chr21	25934047	*JAM2*	587	−1.120553571	0.697781695
cg02992632	chr3	193928074	*FGF12*	8	−1.1105825	0.296886805
cg09137696	chr16	55229916	*MT1A*	163	−1.0697075	0.541279943
cg03192737	chr3	192413893	*OSTN*	877	−1.036060357	0.126389084
cg10031651	chr3	46596690	*LRRC2*	114	−1.033286429	0.101896116
cg05564657	chr3	153014065	*AADAC*	486	−1.018512857	−0.054630151
cg06866657	chr9	124030575	*LHX6*	230	−1.000721071	0.437413369
cg08831744	chr7	45927871	*IGFBP3*	475	1.023873571	0.456738536
cg03462055	chr6	3172555	*TUBB2B*	315	1.175827143	0.725177791
cg00910067	chr19	38409385	*SLC7A10*	837	1.289525714	0.253285976
cg05976074	chr19	38408113	*SLC7A10*	435	1.289525714	0.380324218
cg14546153	chr20	56523721	*FLJ90166*	366	1.331159286	0.43120249
cg00616129	chr12	10718281	*STYK1*	375	1.541281786	−0.209664965
cg23582408	chr20	61600555	*EEF1A2*	394	2.431418571	0.497897542

Note: IllumID represents probe name. Meth.pos stands for the position of methylation site in genome. Distance_to_TSS indicates the distance of methylation site from transcriptional start site of downstream gene. *β* in log_2_⁡FC⁡(β) represents methylation index of methylation probe.

## Additional Points

*Highlights*. (1) We screened 913 DEGs and 8856 methylation sites in LAC tissue samples. (2) In the PPI networks,* MAD2L1*,* AURKB*,* CCNB2*,* CDC20,* and* WNT3A* had higher degrees. (3) There were 29 LAC-associated methylation sites located in 27 DEGs.
